# Cardamonin induces apoptosis of colorectal cancer cells via targeted inhibition of the JAK/STAT3/epithelial-mesenchymal transition (EMT) signaling axis

**DOI:** 10.3389/fphar.2025.1739201

**Published:** 2026-01-08

**Authors:** Min Wu, Chuan Chen, Dan Ren, Zuo Du, Zhenzhong Liu, Wenhu Liu

**Affiliations:** 1 School of Pharmacy, North Sichuan Medical College, Nanchong, China; 2 Innovation Center for Science and Technology, North Sichuan Medical College, Nanchong, China; 3 School of Public Health, North Sichuan Medical College, Nanchong, China

**Keywords:** apoptosis, cardamonin, colorectal cancer, epithelial-mesenchymal transition, JAK/STAT3/epithelial-mesenchymal transition (EMT) signaling axis

## Abstract

**Background:**

Colorectal cancer (CRC) remains a leading cause of cancer-related morbidity and mortality worldwide. Cardamonin (CDN), a bioactive flavonoid derived from the seeds of *Alpinia katsumadai* Hayata, has demonstrated broad-spectrum anticancer potential. However, its specific mechanisms and therapeutic targets in CRC remain poorly elucidated.

**Methods:**

Network pharmacology and molecular docking were employed to identify signaling pathways and targets associated with the anti-CRC activity of CDN. Cell viability, proliferation, migration, and invasion were evaluated using CCK-8, EdU, wound healing, and Transwell assays, respectively. Apoptosis and cell cycle were analyzed by flow cytometry. Proteomic profiling was applied to explore the underlying mechanisms, and the findings were validated using Western blot and functional assays. The antitumor efficacy of CDN *in vivo* was assessed using a subcutaneous xenograft mouse model.

**Results:**

JAK1, STAT3, AKT1, EGFR, IL1B, and ESR1 were identified as shared core targets. The JAK/STAT3 pathway and apoptosis were recognized as pivotal mechanisms mediating the anti-CRC effects of CDN. *In vitro*, CDN inhibited proliferation, migration, and invasion of CRC cells, while promoting apoptosis. Mechanistically, CDN treatment reduced the levels of p-JAK1, p-JAK2, and p-STAT3, indicating inhibition of the JAK/STAT3 pathway. CDN also inhibited the epithelial-mesenchymal transition (EMT) in CRC cells. Consistent with the vitro results, *in vivo*, CDN led to a reduction in the volume and weight of xenograft tumors. It also inhibited the JAK/STAT3 signaling pathway, promoted apoptosis, downregulated Ki-67 expression, and attenuated EMT progression.

**Conclusions:**

CDN inhibits CRC progression and induces apoptosis by targeting the JAK/STAT3/EMT signaling axis, suggesting that CDN is a promising therapeutic agent for CRC.

## 1 Introduction

Colorectal cancer (CRC), a highly prevalent and aggressive gastrointestinal malignancy, is characterized by high incidence, high mortality, and a poor prognosis (Baidoun et al., 2021; Dekker et al., 2019; Eng et al., 2022). Conventional treatments for CRC primarily include surgical resection, endoscopic resection, radiotherapy, and chemotherapy (Li J. et al., 2024; Yan et al., 2025). Chemotherapy plays a pivotal role in eliminating residual CRC cells following local surgery. However, its efficacy is often limited by high rates of chemoresistance and tumor recurrence, thus leading to a 5-year overall survival rate of less than 20% in CRC patients (Baidoun et al., 2021). Therefore, it is imperative to explore synergistic anticancer strategies with multiple mechanisms of action, which could enhance therapeutic efficacy while reducing chemotherapy-associated adverse effects (Abedizadeh et al., 2024; Shin et al., 2023).

Accumulating evidence has established the JAK/STAT3 signaling axis as a pivotal driver of CRC pathogenesis and therapeutic resistance (Li et al., 2023; Ren et al., 2016). Constitutive activation of JAKs sustains persistent STAT3 phosphorylation, which in turn promotes key oncogenic processes, including tumor cell proliferation, survival, metastasis, immune evasion, and chemoresistance (Dinakar et al., 2022; Johnson et al., 2018; Yu et al., 2014). Notably, the JAK2/STAT3 subtype of this pathway is crucial for the maintenance of the stem-like properties in colon cancer cells—an attribute closely linked to therapeutic failure (Prajapati and Kumar, 2024). Despite the development of small-molecule JAK inhibitors such as ruxolitinib and fedratinib, their clinical application remains limited by severe adverse effects, including myelosuppression, immunosuppression, neurological complications, and infections (Coltro and Vannucchi, 2021; Talpaz and Kiladjian, 2021; Verstovsek et al., 2012). Therefore, the identification of safer therapeutic agents that target the JAK/STAT3 signaling axis represents a promising strategy for improving CRC treatment outcomes.

In the search for safer agents, natural products are a prominent source of novel anticancer agents, owing to their structural diversity, generally favorable bioavailability, and low systemic toxicity (Cho et al., 2023; Wang et al., 2018; Xiang et al., 2021). Specifically, cardamonin (CDN), a chalcone isolated from plants in the Zingiberaceae family, has exhibited potent antitumor activity against multiple malignancies, including lung cancer, breast cancer, and esophageal cancer (Nawaz et al., 2020). Mechanistic studies have elucidated its multifaceted actions: in lung cancer, CDN induces the accumulation of reactive oxygen species (ROS), triggering DNA damage and subsequently leading to apoptosis (Makhija et al., 2022); in breast cancer, it suppresses tumor growth by inhibiting HIF-1α-dependent glycolysis (Jin et al., 2019); and in esophageal cancer, CDN promotes apoptosis by inhibiting the PI_3_K/AKT signaling pathway (Wang Y. et al., 2021). Although CDN’s potential in CRC was suggested by a study showing its efficacy in a colitis—associated model via suppression of NF-κB and iNOS (James et al., 2021), its direct targeting of the JAK/STAT3 axis—a key driver of CRC-remains largely unexplored.

In the present study, we aimed to evaluate the therapeutic efficacy of CDN in CRC, focusing on its modulation of the JAK/STAT3 signaling cascade. By integrating network pharmacology, molecular docking, and rigorous *in vitro* and *in vivo* validation, we provide compelling preclinical evidence that CDN suppresses the JAK/STAT3 signaling axis, thereby inhibiting CRC cell proliferation, migration, invasion, and epithelial-mesenchymal transition (EMT). This research not only identifies CDN as a novel dual inhibitor of JAK and STAT3 but also advances the development of low-toxicity, natural product-based therapeutic strategies to overcome chemoresistance and improve CRC management outcomes.

## 2 Materials and methods

### 2.1 Chemicals and reagents

Cardamonin (CDN, purity> 99.5%) was purchased from Lemeitian Medicine (Chengdu, China). The following compounds were obtained from MedChemExpress (Shanghai, China): 5-fluorouracil (5-FU, HY-90006), Ferrostatin-1 (Fer-1, HY-100579), Necrostatin-1 (Nec-1, HY-15760), 3-Methyladenine (3-MA, HY-19312), Z-VAD-FMK (HY-16658B), Stattic (HY-13818), Garcinone D (GarD, HY-N6953), and Upadacitinib (Upa, HY-19569). Assay kits for detecting aspartate aminotransferase (AST), alanine aminotransferase (ALT), blood urea nitrogen (BUN), and creatinine (CREA) were purchased from Nanjing Jiancheng Bioengineering Institute (Nanjing, China). Primary antibodies against CDK1 (19532-1-AP), CDK4 (11026-1-AP), p21 (10355-1-AP), γ-H_2_AX (83307-2-RR), E-cadherin (20874-1-AP), N-cadherin (22018-1-AP), vimentin (10366-1-AP), Ki-67 (28074-1-AP), Bax (50599-2-Ig), Bcl-2 (12789-1-AP), caspase-3 (19677-1-AP), caspase-9 (10380-1-AP), PARP (13371-1-AP), STAT3 (10253-2-AP), GAPDH (60004-1-Ig), and *β*-tubulin (10068-1-AP) were purchased from Proteintech (Wuhan, China). Antibodies against phospho-STAT3 (Tyr705), phospho-JAK1 (Tyr1022/Tyr1023), and phospho-JAK2 (Tyr1007/1008) were obtained from ZenBio (Chengdu, China).

### 2.2 Network pharmacology analysis

Network pharmacology analysis was performed as follows. Potential targets associated with CRC were collected from the GeneCards (https://www.genecards.org/) (Stelzer et al., 2016) and the Online Mendelian Inheritance in Man (OMIM) database (https://omim.org/) (Amberger et al., 2015). Putative targets of the CDN were predicted using the SwissTargetPrediction (https://www.SwissTargetPrediction.ch/) (Daina et al., 2019) and BATMAN-TCM platforms (http://bionet.ncpsb.org.cn/batman-tcm/) (Kong et al., 2024). All acquired targets were combined, and duplicate entries were removed to create a unique target list. The overlapping targets between CDN and CRC were identified using Venny plot. These overlapping targets were then submitted to the STRING database (http://cn.string-db.org/) to construct a protein-protein interaction (PPI) network. The network was built with the following parameters: a minimum interaction score of 0.7, the organism limited to *Homo sapiens*, and disconnected nodes were hidden. The resulting PPI network was imported into Cytoscape (https://cytoscape.org/) for visualization and topological analysis (Shannon et al., 2003). Hub genes were identified using the CytoHubba plugin based on three centrality measures: degree centrality (DC), betweenness centrality (BC), and closeness centrality (CC). Functional enrichment analysis was performed on the overlapping targets. Specifically, Gene Ontology (GO) biological process enrichment was conducted using Metascape (https://metascape.org/) (Zhou et al., 2019), and Kyoto Encyclopedia of Genes and Genomes (KEGG) pathway enrichment was carried out with the Database for Annotation, Visualization and Integrated Discovery (DAVID) (https://davidbioinformatics.nih.gov/) (Sherman et al., 2022). The top 15 significantly enriched biological processes and KEGG pathways were visualized using the online platform bioinformatics. com.cn (https://www.bioinformatics.com. cn/).

### 2.3 Molecular docking analysis

Three-dimensional (3D) crystal structures of JAK1 (PDB ID: 6AAH), JAK2 (PDB ID: 7F7W), and STAT3 (PDB ID: 6NUQ) were obtained from the Research Collaboratory for Structural Bioinformatics (RCSB) Protein Data Bank (https://www.rcsb.org/). The molecular structure of CDN was retrieved from the PubChem database (https://pubchem.ncbi.nlm.nih.gov/). To prepare the proteins for docking, water molecules and co-crystallized ligands were removed from the protein structures. The grid box parameters, including the central coordinates and dimensions, were defined based on the predicted binding sites of each protein-ligand complex to encompass the potential interaction region. Molecular docking simulations were performed using AutoDock Vina (http://vina.scripps.edu/) (Trott and Olson, 2010) to dock CDN against JAK1, JAK2, and STAT3. The binding pose with the lowest predicted binding energy for each protein was selected for further analysis. Hydrogen bonding interactions between CDN and the key residues of the target proteins were analyzed using PyMOL to elucidate the binding mode and identify critical interaction sites.

### 2.4 Cell culture

Human CRC cell lines (HCT116, RKO, and SW620) and the normal colonic mucosal epithelial cell line NCM460 were obtained from the Shanghai Cell Bank, Chinese Academy of Sciences (Shanghai, China). The CRC cell lines were cultured in Dulbecco’s modified Eagle’s medium (DMEM) (Keygen Biotechnology Co., Ltd., Nanjing, China), supplemented with 10% fetal bovine serum (FBS, Gibco, Oklahoma, United States). NCM460 cells were maintained in RPMI 1640 medium containing 10% FBS. All cells were routinely cultured in a humidified incubator with 5% CO_2_ at 37 °C.

### 2.5 Cell viability assay

Cell viability was assessed using the Cell Counting Kit-8 (CCK-8, Beyotime, China, Cat. No. C0037) according to the manufacturer’s instructions. Briefly, cells were seeded in 96-well plates at a density of 5 × 10^3^ cells per well. After 24 h, the cells were treated with a gradient of CDN concentrations (0, 1, 2, 4, 8, 16, 32, and 64 µM) for an additional 48 h. Subsequently, CCK-8 was added to each well, and the plates were incubated at 37 °C for 2 h. The optical density (OD) at 450 nm was measured using a microplate reader.

### 2.6 Colony formation assay

Cells were seeded into 6-well plates at a density of 1 × 10^3^ cells per well. After 24 h of incubation, the cells were treated with CDN (0, 4, and 8 μM) for 48 h. Subsequently, the medium was replaced with CDN-free fresh medium, and the cells were cultured for an additional 14 days to allow colony formation. The resulting colonies were fixed with 4% paraformaldehyde for 20 min and stained with 0.1% crystal violet for 20 min. The number of visible colonies was manually counted.

### 2.7 Cell proliferation assay

Cells were seeded in 24-well plates at a density of 5 × 10^4^ cells per well. After adherence, the cells were treated with CDN (0, 8, and 16 μM) for 48 h 5-ethynyl-2’-deoxyuridine (EdU) was then added to the culture medium, and incubation continued for additional 4 h. Thereafter, cells were fixed with 4% paraformaldehyde for 20 min and permeabilized with 0.1% Triton X-100. The Click-iT reaction was performed to detect incorporated EdU following the manufacturer’s protocol. Cell nuclei were counterstained with Hoechst 33,342 for 10 min. Fluorescence images were captured using an Olympus FV3000 confocal microscope (Olympus Corporation, Japan), and the ratio of EdU-positive cells to total Hoechst-positive cells was calculated.

### 2.8 Calcein AM/PI double staining assay

Cell viability and mortality were assessed using a Calcein-AM/PI double staining kit (Beyotime, China, Cat. No. C2015) according to the manufacturer’s instructions. Cells were seeded in 35-mm dishes at a density of 1 × 10^5^ cells per plate. After adherences, the cells were treated with CDN (0, 8, and 16 μM) for 48 h. Following treatment, the cells were incubated with a Calcein AM/PI solution at 37 °C for 30 min in the dark. The cells were then gently washed with PBS to remove excess dye. Live cells and dead cells were observed and imaged using an Olympus FV3000 confocal microscopes.

### 2.9 Lactic dehydrogenase (LDH) release assay

Lactic dehydrogenase release levels were measured using the Lactic Dehydrogenase Release Assay Kit (Cat. No. C0016, Beyotime, China) according to the manufacturer’s instructions. Briefly, cells were seeded in 96-well plates at a density of 5 × 10^3^ cell per. After 24 h, the cells were treated with various concentrations of CDN for 48 h. After treatment, the supernatant from each well was carefully collected and transferred to a new 96-well plate. The LDH detection reagent was added to the supernatant, and the mixture was incubated on a shaker at room temperature for 30 min protected from light. The absorbance was measured at 490 nm using a microplate reader.

### 2.10 Mitochondrial membrane potential (MMP) assay

MMP assays were performed according to our previously described protocols (Liu et al., 2025). Cells were seeded in 6-well plates (1 × 10^5^ cells per well) for 24 h. After treatment with CDN for 48 h, cells were harvested, washed with PBS. The cell suspension was incubated with JC-1 staining solution at 37 °C for 20 min, washed twice, and analyzed by flow cytometry (Sony SA3800). The MMP was quantified by the ratio of red fluorescence to green fluorescence.

### 2.11 Reactive oxygen species (ROS) assay

Intracellular ROS levels were quantified using a Reactive Oxygen Species Assay Kit (Cat. No. S0033, Beyotime, China) following the manufacturer’s protocols. Briefly, cells were seeded into 6-well plates at a density of 1 × 10^5^ cells per well and allowed to adhere overnight. Subsequently, cells were treated with CDN at the specified concentrations for 48 h. After treatment, cells were incubated with 10 μM DCFH-DA at 37 °C in the dark for 20 min. Following two washes with serum-free medium to remove excess probe, the fluorescence intensity was measured using a Sony SA3800 spectral cell analyzer.

### 2.12 Apoptosis assay

Apoptosis was assessed using an Annexin V-FITC assay kit (Beyotime, China, Cat. No. C1062) as described previously (Nong et al., 2022). Briefly, cells were seeded in 6-well plates at a density of 1 × 10^5^ cells per well and treated with CDN at various concentrations for 48 h. Thereafter, the cells were washed with PBS and resuspended in binding buffer. The suspensions were then incubated with Annexin V-FITC and propidium iodide (Keygen Biotech, Nanjing, China) for 15 min at the room temperature in the dark. Apoptosis was analyzed using a Sony SA3800 spectral cell analyzer.

### 2.13 Scratch assay

Cell migration was assessed using the scratch assay according to our previously described protocols (Liu et al., 2025). Briefly, cells were seeded into 6-well plates at a density of 5 × 10^5^ cells per well and cultured until full confluence. A straight scratch was created in the cell monolayer using a sterile 200 µL pipette tip. After washing with PBS to remove detached cells, serum-free medium containing varying doses of CDN was added. The plates were incubated and images of the scratches were captured at predefined time points. The migration ability was evaluated by measuring the rate of wound closure.

### 2.14 Transwell assay

Transwell assays were conducted according to our previously described protocols (Liu et al., 2025). Cells were resuspended in serum-free medium containing various concentrations of CDN and seeded into the upper chamber of Matrigel-precoated Transwell inserts at a density of 1 × 10^5^ cells per well. The lower chamber was filled with medium supplemented with 10% FBS as a chemoattractant. After 48 h of incubation, non-invading cells on the upper surface of the membrane were gently removed with a cotton swab. Invading cells on the lower surface were fixed with 4% paraformaldehyde, stained with 0.1% crystal violet, and quantified by counting six random fields per membrane under a microscope.

### 2.15 Cell cycle assay

Cell cycle assays were performed using a Cell Cycle Detection Kit (KGA512, Keygen, Nanjing, China) following the manufacturer’s protocols. Cells were seeded in 6-well plates at a density of 2 × 10^5^ cells per well for 24 h and then treated with CDN for 48 h. Cells were harvested, fixed in 70% cold ethanol, and stored at 4 °C overnight. Prior to analysis, the fixed cells were washed with PBS, and stained with PI/RNase A buffer for 30 min in the dark. Cell cycle distribution was determined by analyzing DNA content via flow cytometry (Sony SA3800).

### 2.16 Quantitative proteomic analysis of CDN-treated CRC cells

#### 2.16.1 Protein extraction and trypsin digestion

HCT116 cells were treated with 16 μM CDN for 48 h. Protein extraction, peptide preparation, and fractionation were performed as previously described (Liu et al., 2018; Liu et al., 2023). Briefly, cells were lysed with lysis buffer and the supernatant was collected as whole-cell lysate. For each sample, 100 μg protein was reduced with 10 mM dithiothreitol at 56 °C for 30 min, alkylated with 10 mM iodoacetamide for 30 min, and digested using the filter-aided proteome preparation (FASP) method with trypsin at 37 °C. Peptides were collected by centrifugation (14,000×g, 10 min), desalted using C18 Stage Tips, and dried in a vacuum concentrator. Peptide fractionation was performed using a homemade reverse-phase C18 pipette tip column with a stepped acetonitrile gradient, generating nine fractions. Quality control was ensured by including 293T cell lysates to monitor LC-MS/MS system performance.

#### 2.16.2 LC-MS/MS analysis

Peptides were resuspended in 0.1% formic acid and analyzed with Orbitrap Fusion Lumos mass spectrometer (Thermo Fisher Scientific,United States) equipped with an Easy-nLC 1200 high-performance liquid chromatography (HPLC) system (Thermo Fisher Scientific, United States). Peptides were subsequently loaded onto a homemade trap column (3 μm particle size, 120 Å pore size, 100 μm × 2.0 cm, SunChrom, United States) and separated on a homemade analytical microcolumn (1.9 μm particle size, 120 Å pore size, 150 μm × 15.0 cm, SunChrom, United States). The separation employed a 60-min linear gradient of mobile phase B from 7% to 40% mobile phase B (0.1% formic acid in acetonitrile) at a constant flow rate of 600 nL/min. The gradient profile was as follows: 7%–10% B for 3 min, 10%–25% B for 39 min, 25%–40% B for 11 min, 40%–95% B for 1 min, and 95% B held for 6 min.

Mass spectrometry analysis was performed in a data-dependent acquisition (DDA) mode. Full MS scans were acquired at a resolution of 120,000 with an automatic gain control (AGC) target of 5e5. A top-speed mode was used with a 3-s cycle time. The most intense precursor ions were isolated by the quadrupole with a 1.6Th window. Higher-energy collision dissociation (HCD) was performed with a normalized collision energy (NCE) of 32%. The fragment ions were detected at a resolution of 15,000 with an MS^2^ AGC target of 5e4. A dynamic exclusion was set to 30 s.

#### 2.16.3 Proteomic data analysis

MS data were analyzed by searching against the human NCBI RefSeq protein database using the Mascot 2.3 search engine (Matrix Science Inc.). Precursor and product ion mass tolerances were set to 20 ppm and 0.5 Da, respectively, with a maximum of two missed cleavages permitted. The protein-level false discovery rate (FDR) was strictly constrained to 1%. For proteome profiling, fixed modifications included carbamidomethylation of cysteine, while variable modifications comprised N-terminal acetylation and oxidation of methionine. Protein quantification was performed using intensity-based absolute quantification (iBAQ) (Fu et al., 2020; Lai et al., 2016; Yuan et al., 2021). To normalize protein abundance across samples, the fraction of total (FOT) was calculated as the ratio of a protein’s iBAQ value to the total iBAQ of all identified proteins in the same sample; all FOT values were scaled by 10^5^ to improve data visualization. Only proteins with at least 50% valid values in each group were included for further analysis. Missing values for these proteins were imputed using the K-Nearest Neighbors algorithm via an R package. For differential abundance analysis, proteins in the CDN-treated group were considered significantly differentially abundant compared to the control group if they exhibited a fold change of ≥1.5 (either increase or decrease) with a p-value <0.05. Gene Ontology (GO) enrichment analysis for biological processes was performed using the Metascape database. GO terms meeting the criteria of p<0.05 and enriched with at least three proteins were clustered by membership similarity, and the top 15 most significantly enriched terms were visualized. For PPI network construction, interactions were inferred via the STRING database. Pathway enrichment analysis was performed using the KEGG database.

### 2.17 Western blot assay

Western blot assays were performed according to our previously described methods (Liu et al., 2025; Wu et al., 2025). Briefly, protein samples were homogenized and lysed in lysis buffer to extract total proteins. After quantifying the protein concentration, equal amounts of protein were separated by SDS-PAGE and transferred to a polyvinylidene fluoride (PVDF) membrane. The membrane was blocked with 5% skim milk for 1 h, followed by overnight incubation with primary antibodies specific to the target proteins at 4 °C with agitation. After thorough washing with TBST, horseradish peroxidase-conjugated secondary antibodies were added and incubated for 1 h at room temperature. Protein bands were visualized using an ECL detection system. Band intensities were quantified by analyzing grayscale values with ImageJ software, and relative protein expression levels were normalized to internal control proteins to correct for loading variations.

### 2.18 Histopathological examination and immunohistochemical staining

Tumor tissues harvested from mice were fixed in 10% formalin and subsequently embedded in paraffin. After embedded, tissue sections were deparaffinized and stained with hematoxylin and eosin (H&E). Immunohistochemical staining was then performed using previously established protocols (Liu et al., 2022a; Ruan et al., 2024).

### 2.19 Subcutaneous xenograft model and biosafety assessment

Animal experiments were performed as described in our previous methods (Liu et al., 2025). Fifty female BALB/c nude mice, aged 4–5 weeks, were obtained from Beijing Vital River Laboratory Animal Technology Co., Ltd. (Beijing, China). They were housed in a specific pathogen-free facility with controlled conditions (temperature: 25 °C ± 2 °C, humidity: 50% ± 5%), a 12-h light/dark cycle, and ad libitum access to food and water. The experimental procedures and animal care protocols were approved by the Animal Ethics Committee of North Sichuan Medical College (Approval No.: NSMC 2025062). After a 5-day acclimation period, HCT116 cells (5 × 10^6^ cells suspended in 0.2 mL PBS) were injected subcutaneously into the right axillary region of each mouse. Once the average tumor volume reached 50 mm^3^, mice were randomly assigned to four groups (n = 6 per group): control group (daily intraperitoneal injection of saline), low-dose CDN group (daily intraperitoneal injection of 10.0 mg/kg CDN), high-dose CDN group (daily intraperitoneal injection of 20.0 mg/kg CDN), and 5-FU group (intraperitoneal injection of 5-FU at 20.0 mg/kg every other day). Body weight and tumor volume were measured every 2 days. After 14 days of treatment, mice were anesthetized using 2% isoflurane inhalation. Blood samples were collected, and plasma was prepared by centrifugation at 4 °C for 10 min. Tumor tissues and vital organs (heart, liver, spleen, lung, and kidney) were excised and weighed. Tumor volume was calculated using the formula: Volume = (length × width^2^)/2. The biosafety assessment of CDN included evaluating hepatic and renal function through plasma levels of AST, ALT, CREA, and BUN. It also assessed CDN-induced pathological changes in the heart, liver, spleen, lung, and kidney using H&E staining.

### 2.20 Blood sample and hemolysis assay

Blood was collected from mice into EDTA-coated tubes, and plasma was subsequently prepared according to an established protocol (Liu et al., 2022b). For the hemolysis assay, whole blood samples were centrifuged at 3000×*g* for 10 min to isolate erythrocytes. After being washed twice with PBS, the erythrocytes were resuspended in PBS to prepare a 4% (*v/v*) suspension. Then, 40 µL of this erythrocyte suspension was mixed with 1 mL of serially diluted CDN solutions (25, 50, 100, 200, 400, 800, and 1600 μg/mL). For the control groups, 40 μL of the same erythrocyte suspension was mixed with 1 mL of normal saline (negative control) or distilled water (positive control), respectively. All mixtures were incubated at 37 °C for 2 h in a humidified incubator, followed by centrifugation at 3,000×*g* for 10 min. The optical density of the supernatant was measured at 545 nm using a spectrophotometer, and the hemolysis rate was calculated using the formula: Hemolysis (%) = [(OD_sample- OD_negative control)/(OD_positive control - OD_negative control)] × 100%.

### 2.21 Statistical analysis

Statistical analysis was completed using SPSS Statistics Software (v23.0). GraphPad Prism (v8.0.1) was used for data visualization. All data were presented as means ± standard error of the mean (SEM) from at least three independent experiments. The significance of differences between groups was determined using one-way analysis of variance and Student’s t-test. A p-value <0.05 was considered statistically significant.

## 3 Results

### 3.1 Network pharmacology identifies the JAK/STAT3 signaling pathway as a core therapeutic target of CDN

**FIGURE 1:**
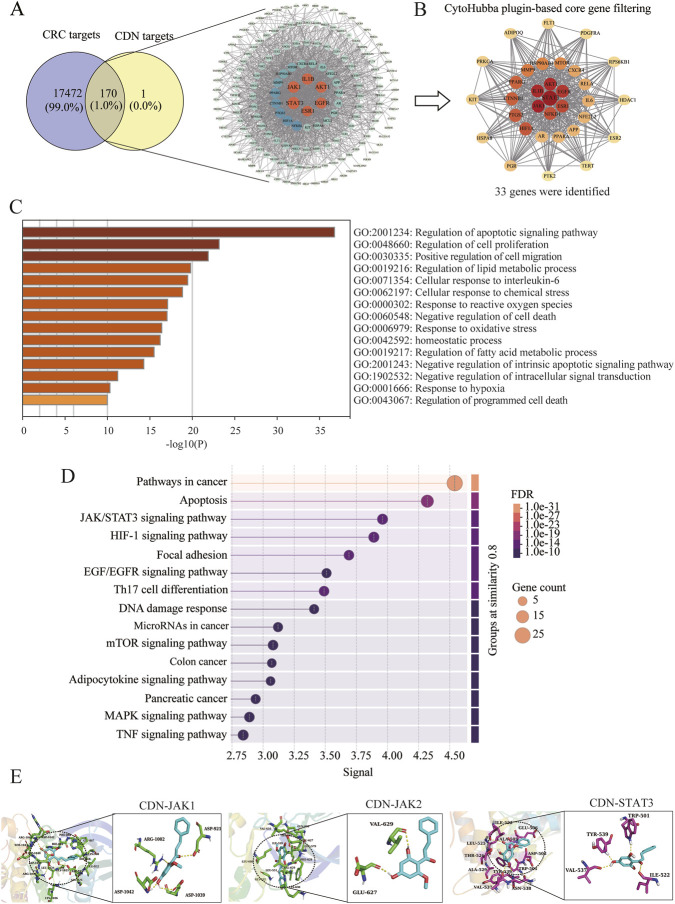
Network pharmacology and molecular docking identify the core targets and pathways of CDN in CRC treatment **(A)** Venn diagram and PPI network of overlapping targets between CDN and CRC. **(B)** Identification of the core targets using the CytoHubba plugin in Cytoscape. **(C)** Gene Ontology enrichment analysis of biological processes associated with the overlapping targets. **(D)** KEGG pathway enrichment analysis of the core targets. **(E)** Molecular docking analysis predicting the binding modes of CDN with JAK1, JAK2, and STAT3.

Potential targets of CDN were predicted using the SwissTargetPrediction and BATMAN-TCM databases, yielding 171 candidates. Concurrently, 17,642 CRC-related targets were retrieved from the GeneCards and OMIM databases (Figure 1A). Intersection analysis identified 170 overlapping targets common to both CDN and CRC. A CDN-CRC target network was constructed using Cytoscape based on these 170 targets (Figure 1A). PPI network analysis was then conducted with the CytoHubba plugin, and 33 hub genes were identified based on topological features (Figure 1B). Among these, STAT3, JAK1, IL1B, AKT1, EGFR, ESR1, and NFKB1 showed high connectivity and centrality, suggesting their core regulatory roles.

Functional enrichment analysis of the 170 overlapping targets was performed. GO terms were significantly enriched in biological processes such as regulation of apoptotic signaling, cell proliferation, lipid metabolism, and response to reactive oxygen species (Figure 1C). KEGG pathway analysis revealed the top 15 enriched pathways, most of which were associated with tumor development. Among these, pathways in cancer, apoptosis, and the JAK-STAT signaling pathway showed the highest enrichment scores (Figure 1D). These results suggest that the anti-CRC effect of CDN is primarily mediated through modulation of the JAK/STAT signaling pathway and its influence on apoptosis.

To further investigate the interaction between CDN and key components of the JAK/STAT3 pathway, molecular docking simulations were carried out using AutoDock Vina. The results indicated that CDN exhibited strong binding affinity for JAK1, JAK2, and STAT3. The calculated binding free energy (*Δ*G) values were −6.86 ± 0.34 kcal/mol (pKi = 5.03 ± 0.26) for JAK1, -6.75 ± 0.46 kcal/mol (pKi = 4.95 ± 0.33) for JAK2, and -6.79 ± 0.55 kcal/mol (pKi = 4.98 ± 0.41) for STAT3, respectively (Figure 1E). Further analysis of the binding modes using PyMOL revealed that CDN forms hydrogen bonds with key residues in each protein: Arg-1002, Asp-1039, and Asp-1042 in JAK1; Glu-627 and Val-629 in JAK2; and Tyr-539, Trp-501, and Val-537 in STAT3 (Figure 1E).

### 3.2 Multi-dimensional analysis identifies the JAK/STAT3 pathway as a therapeutic target in colorectal cancer

**FIGURE 2:**
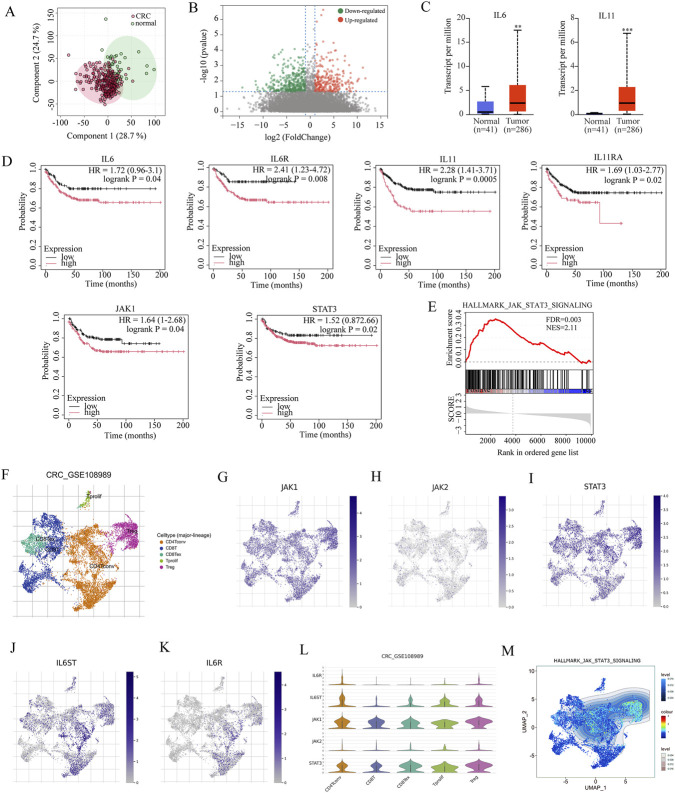
Multi-dimensional analysis identifies the JAK/STAT3 signaling axis as a potential therapeutic target for CRC **(A)** PCA of gene expression profiles from CRC tissues and their adjacent normal tissues. **(B)** A volcano plot of the 3838 most variable genes from TCGA database. **(C)** mRNA expression levels of IL6and IL11in CRC versus normal tissues from TCGA and GTEx databases. **(D)** Overall survival analysis of patients stratified by expression of IL6, IL6R, IL11, IL11RA, JAK1, and STAT3. **(E)** GSEA showing significant activation of the JAK/STAT3 pathway in CRC tissue. **(F)** Uniform Manifold Approximation and Projection (UMAP) plot of 11125 single cells from 12 CRC patients (GSE108989 dataset), colored by cell type. **(G–K)** UMAP visualizations of JAK1, JAK2, STAT3, IL6ST, and IL6Rexpression in single cells. **(L)** Quantitative analysis of JAK1, JAK2, STAT3, IL6ST, and IL6R expression across different cell type. **(M)** Pathway enrichment analysis based on gene signatures derived from single-cell RNA sequencing.

We performed comparative transcriptomic analysis using mRNA expression profiles from 286 primary CRC tissues and 41 adjacent normal colon tissues obtained from The Cancer Genome Atlas (TCGA) (Figures 2A,B). The analysis revealed significant upregulation of IL6 and IL11 in CRC tissues compared with normal samples (Figure 2C). Overall survival (OS) analysis of the CRC cohort indicated that high expression of IL6, IL6R, IL11, IL11RA, JAK1, and STAT3 was associated with significantly poorer survival outcomes (Figure 2D). Gene Set Enrichment Analysis (GSEA) further confirmed strong activation of the JAK/STAT3 signaling pathway in CRC tissues (Figure 2E).

To further investigate the cellular-level expression patterns, we analyzed single-cell RNA sequencing (scRNA-seq) data from 12 CRC patients in the GSE108989 dataset (Figure 2F). The results showed significantly elevated expression of JAK1, STAT3, IL6ST, and IL6R, while JAK2 expression remained unchanged (Figures 2G–L). GSEA based on single-cell-derived gene signatures also indicated strong activation of the JAK/STAT3 signaling axis in CRC, consistent with the TCGA cohort results (Figure 2M).

Together, these multi-dimensional analyses provide compelling evidence that the JAK/STAT3 signaling pathway is aberrantly hyperactivated in CRC. These findings clarify the pathogenic role of this pathway in CRC progression and highlight its potential as a therapeutic target.

### 3.3 CDN exhibited selective cytotoxicity against CRC cells with minimal effects on normal colonic mucosal epithelial cells

**FIGURE 3:**
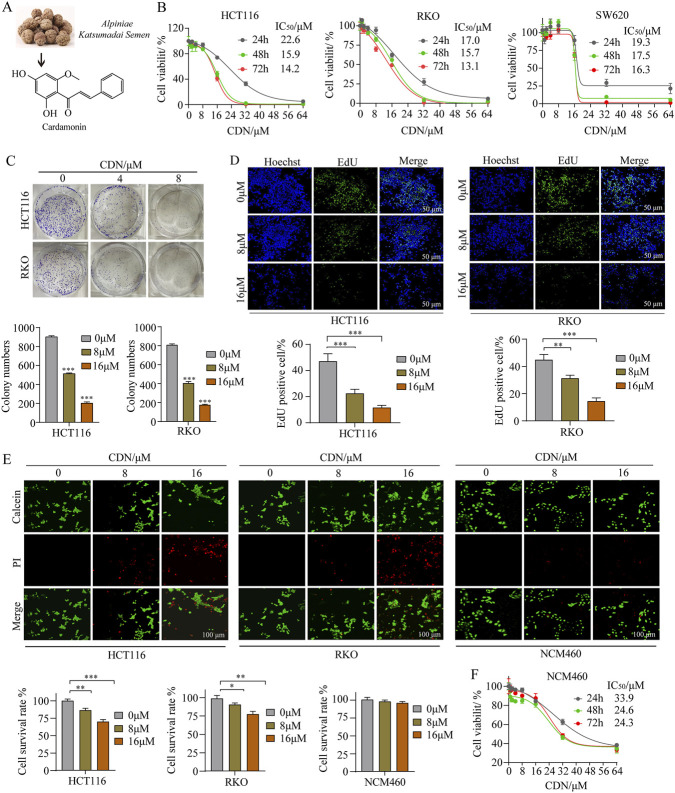
Cytotoxic effect of CDN on colorectal cancer cells. **(A)** Chemical structure and origin of CDN. **(B)** Cell viability of CRC cells was determined using the CCK-8 assay after 48 h of CDN treatment. **(C)** Colony formation ability was evaluated via colony formation assays. **(D)** Cell proliferation was assessed using EdU staining after CDN treatment. **(E)** Calcein AM/PI dual staining was performed to distinguish live and dead cells. Scale bar = 200 μm. **(F)** NCM460 cells were treated with CDN for 24, 48, and 72 h, and cell viability was measured using CCK-8 assays. All data were presented as the means ± SEM, *n* = 3. ^*^ *p* < 0.05, ^**^ *p* < 0.01, ^***^ *p* < 0.001, vs. control group.

The chemical structure of CDN is shown in Figure 3A. We evaluated the anti-proliferative activity of CDN by treating three human CRC cell lines (HCT116, RKO, and SW620) with increasing concentrations (0–64 μM). CCK-8 assays showed that CDN inhibited cell viability in a dose- and time-dependent manner (Figure 3B). The half-maximal inhibitory concentration (IC_50_) values at 24, 48, and 72 h were as follows: 22.6, 15.9, and 14.2 μM for HCT116; 17.0, 15.7, and 13.1 μM for RKO; and 19.3, 17.5, and 16.3 μM for SW620. Given their relatively higher sensitivity, HCT116 and RKO cells were selected for subsequent experiments. Consistent with the CCK-8 results, colony formation assays confirmed that CDN significantly reduced the clonogenic ability of both cell lines (Figure 3C). EdU incorporation assays further validated the anti-proliferative effect of CDN (Figure 3D).

To assess the selectivity of CDN, we compared its effects on CRC cells and the normal colonic epithelial cell line NCM460 using Calcein-AM/PI double staining. CDN exhibited strong cytotoxicity against CRC cells but had negligible effects on NCM460 cells (Figure 3E). The IC_50_ values of CDN in NCM460 cells at 24, 48, and 72 h were 33.9, 24.6, and 24.3 μM, respectively (Figure 3F). The corresponding selectivity indices (SI) for CDN were calculated: for HCT116 cells, the SIs were 1.5 (24 h), 1.55 (48 h), and 1.71 (72 h); for RKO cells, 1.99 (24 h), 1.57 (48 h), and 1.85 (72 h); and for SW620 cells, 1.76 (24 h), 1.41 (48 h), and 1.49 (72 h). Collectively, these results demonstrate that CDN selectively inhibits the proliferation of CRC cells while showing minimal toxicity to normal colonic epithelial cells.

### 3.4 CDN suppresses migration, invasion, and EMT in CRC cells

**FIGURE 4:**
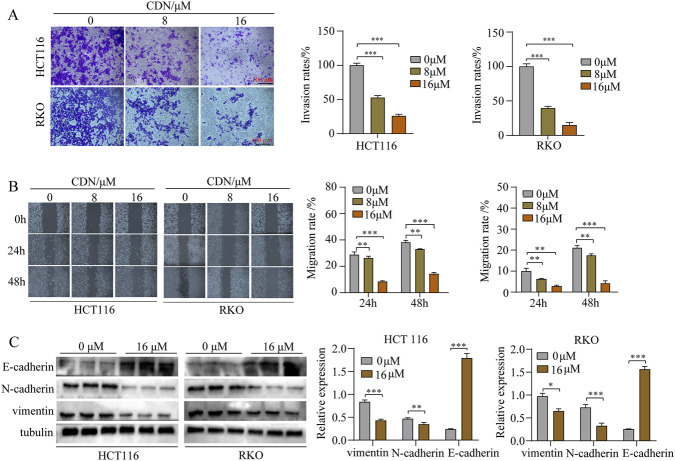
CDN inhibits migration, invasion, and the epithelial-mesenchymal transition process of CRC cells. **(A)** Cellular invasive capacity was assessed via the Transwell invasion assay. **(B)** The migration capacity of CRC cells was evaluated using the wound-healing assay. **(C)** Expression of E-cadherin, N-cadherin, and vimentin was detected by Western blot. All data are presented as the mean ± SEM, *n* = 3, ^*^ *p* < 0.05, ^**^ *p* < 0.01, ^***^ *p* < 0.001, vs. control group.

Cell migration and invasion are critical drivers of tumor progression and malignant transformation (Keleg et al., 2003; Novikov et al., 2021). To investigate whether CDN influences these processes in CRC, we performed wound-healing and Transwell invasion assays. The results showed that CDN treatment significantly suppressed the migration and invasion of HCT116 and RKO cells in a dose-dependent manner (Figures 4A,B). To explore the underlying mechanism, we examined the expression of EMT-related markers by Western blot. CDN treatment increased the expression of E-cadherin and decreased the levels of N-cadherin and vimentin in both cell lines (Figure 4C), indicating that CDN inhibits migration, invasion, and EMT in CRC cells.

### 3.5 CDN reduces mitochondrial membrane potential, elevates ROS levels, and induces cell cycle arrest in CRC cells

**FIGURE 5:**
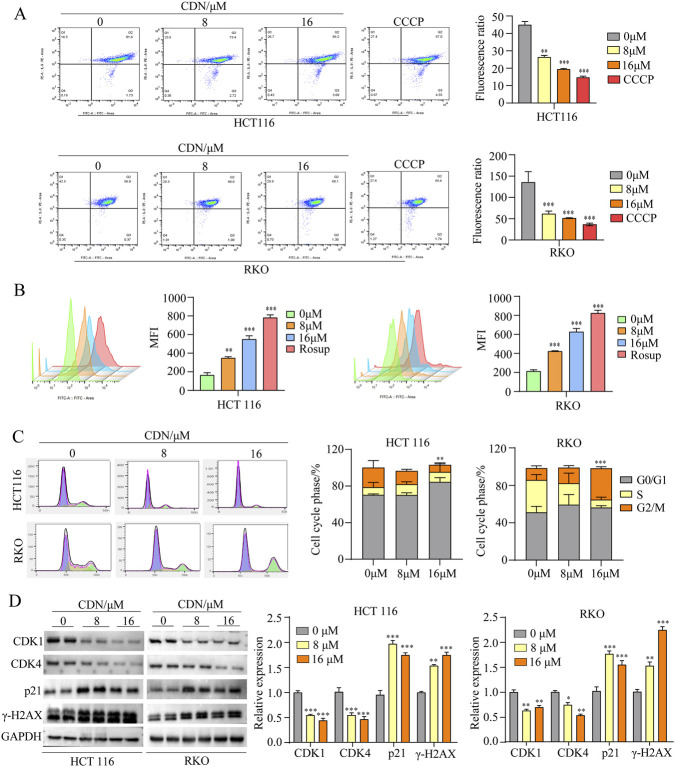
CDN induced elevated ROS levels, mitochondrial impairment, and cell cycle arrest in CRC cells. **(A)** Intracellular ROS levels were quantified in CRC cells using flow cytometry. **(B)** Mitochondrial membrane potential was measured in CRC cells using flow cytometry. **(C,D)** Cell cycle distribution was analyzed using a cell cycle kit, and Western blotting analysis was employed to quantify the expression levels of cell cycle-associated proteins. *n* = 3, ^*^ *p* < 0.05, ^**^ *p* < 0.01, ^***^ *p* < 0.001, vs. control group.

**FIGURE 6:**
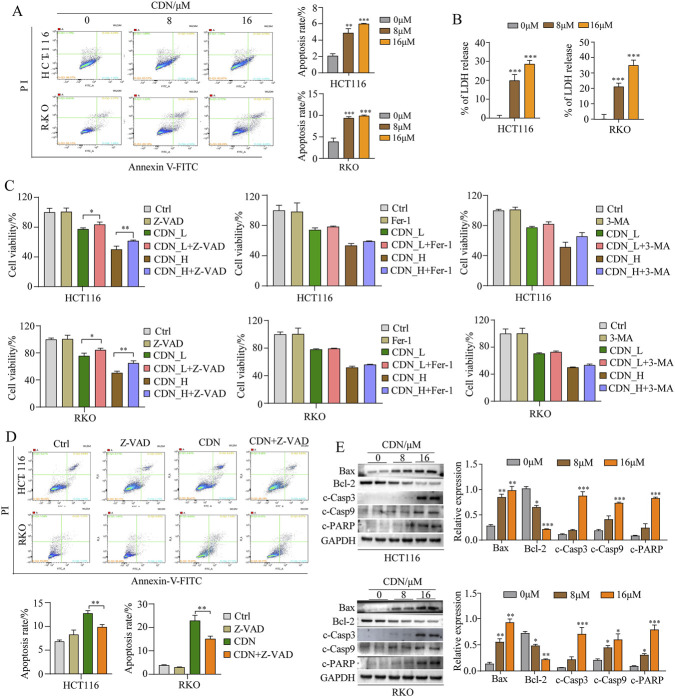
CDN-induced apoptosis in CRC cells relies on the caspase signaling pathway. **(A)** Determination of apoptotic cell using Annexin V-FITC/PI double staining combined with flow cytometry. **(B)** Determination of lactic dehydrogenase (LDH) release using the LDH assay Kit. **(C)** Cell viability was measured using the CCK-8 Kit after 48-h treatment with Z-VAD, CDN, or Z-VAD + CDN. **(D)** Cell apoptosis was analyzed after treatment with Z-VAD, CDN or CDN + Z-VAD, respectively. **(E)** Western blot was used to analyze the expression of Bax, Bcl-2, cleaved caspase-3 (c-casp3), cleaved caspase-9 (c-casp9), and cleaved PARP (c-PARP) in HCT116 and RKO cells. *n* = 3, ^*^ *p* < 0.05, ^**^ *p* < 0.01, ^***^ *p* < 0.001, vs. control group.

Mitochondrial membrane potential (MMP) dysfunction, characterized by loss of MMP and accumulation of reactive oxygen species (ROS), is a hallmark of mitochondria-mediated apoptosis and a well-established mechanism of action for many antitumor agents (Liu et al., 2025; Yang et al., 2024; Yevale et al., 2025). To determine whether CDN affects mitochondrial function, we measured MMP in HCT116 and RKO cells using JC-1 staining. The results showed that CDN treatment induced a significant, dose-dependent reduction in MMP (Figure 5A). Quantitative analysis showed that compared with controls, 8 μM and 16 μM CDN decreased MMP by 41.4% and 56.8% in HCT116 cells, and by 54.6% and 62.1% in RKO cells, respectively. Given the consistency between these results and the apoptotic phenotypes observed in our Annexin V/PI staining assays (Figure 6A), we conclude that CDN-induced MMP loss is a key event in triggering apoptotic signaling in CRC cells.

ROS accumulation not only exacerbates mitochondrial impairment but also directly triggers apoptosis by damaging cellular components and activating pro-apoptotic signaling cascades (Wang Z. et al., 2021). Given the close reciprocal relationship between mitochondrial dysfunction and ROS generation, we next assessed intracellular ROS levels. Flow cytometry analysis showed a concentration-dependent increase in ROS production in both cell lines following CDN treatment (Figure 5B). This elevated ROS level, in turn, exacerbates mitochondrial damage and promotes apoptosis-a finding consistent with the observed MMP loss.

Many anticancer agents exert their anti-proliferative effects by disrupting the cell cycle (Li et al., 2025). Moreover, ROS overproduction is a well-documented cause of cell cycle arrest, as it modulates DNA damage responses and regulates key cycle-regulatory proteins (Mackova et al., 2024; Sahoo et al., 2022). To determine whether CDN causes cell cycle arrest, we investigated its effect on cell cycle distribution. Flow cytometric analysis showed that CDN induced cell cycle arrest with distinct patterns between the 2 cell lines: HCT116 cells were arrested primarily in the G_0_/G_1_ phase, whereas RKO cells accumulated in the G_2_/M phase (Figure 5C). Corroborating these findings, Western blot analysis demonstrated that CDN treatment upregulated γ-H2AX and p21, and downregulated CDK1 and CDK4 (Figure 5D). These results suggest that CDN-induced ROS triggers DNA damage (as marked by γ-H2AX), leading to p21 upregulation, which subsequently mediates cell cycle arrest by inhibiting CDK1/4.

### 3.6 CDN induces caspase-dependent apoptosis in CRC cells

Apoptosis plays a critical role in cancer cell survival, making it a key target for novel anticancer drug discovery (An et al., 2019). Therefore, to elucidate the mechanism underlying the anti-CRC activity of CDN, we first examined apoptotic cell death using Annexin V/PI staining and flow cytometry. As shown in Figure 6A, CDN treatment resulted in a significant, dose-dependent increase in the percentage of apoptotic cells in both CRC cell lines. Consistent with these findings, the release of lactic dehydrogenase (LDH)-a marker of plasma membrane integrity loss-was also elevated in a concentration-dependent manner (Figure 6B). To identify the specific cell death pathway involved, HCT116 and RKO cells were pretreated with 10 μM of inhibitors targeting different death modalities: the pan-caspase inhibitor Z-VAD-FMK (apoptosis), Nec-1 (necroptosis), 3-MA (autophagy), and Fer-1 (ferroptosis). Strikingly, only Z-VAD significantly attenuated the inhibitory effect of both low- and high-dose CDN on the viability of HCT116 and RKO cells (Figure 6C), whereas the other inhibitors showed no significant effect. Moreover, Z-VAD effectively reversed CDN-induced apoptotic cell death, as shown by a marked reduction in the apoptotic population (Figure 6D).

We next examined the expression of key apoptotic regulators by Western blot. CDN treatment increased the expression of pro-apoptotic Bax, decreased anti-apoptotic Bcl-2, and enhanced the levels of cleaved caspase-3, cleaved caspase-9, and cleaved PARP in both cell lines (Figure 6E). Taken together, these results demonstrate that CDN induces cell death in CRC cells primarily through a caspase-dependent apoptotic pathway.

### 3.7 Proteomic profiling of CDN-treated HCT116 cells

**FIGURE 7:**
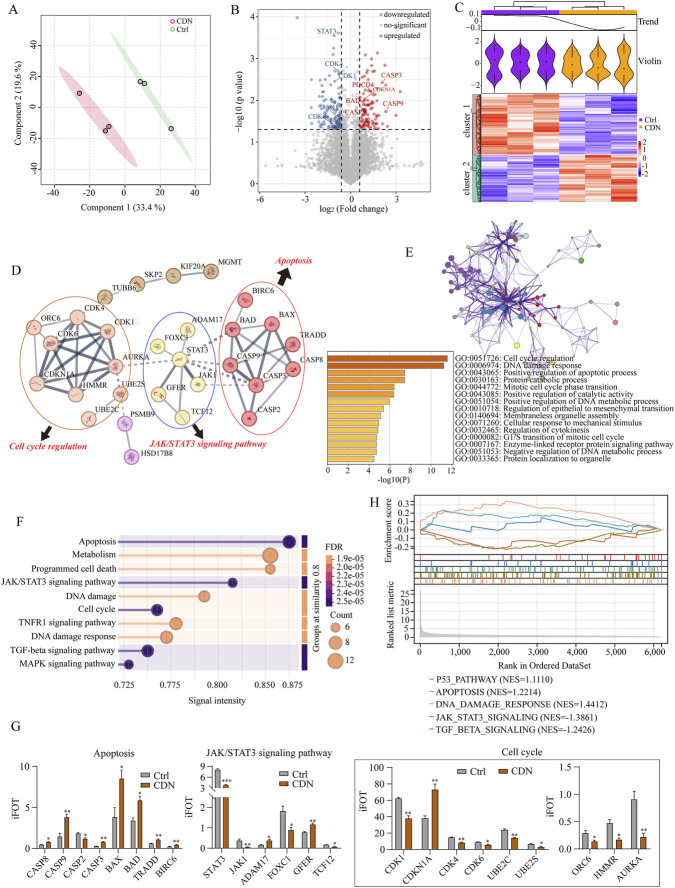
Proteomic profiling of CDN-treated HCT116 cells. **(A)** Principal component analysis (PCA) was performed to compare the proteomic profiles of untreated and CDN-treated HCT116 cells. **(B)** Volcano plot analysis was used to identify differentially expressed proteins. **(C)** A heatmap visualized the expression trends of the identified differentially expressed proteins. **(D)** Protein-protein interaction (PPI) analysis was conducted to explore the potential interaction networks among the identified differentially expressed proteins. **(E)** Network plots and corresponding p-values were used to present the results of Gene Ontology (GO) enrichment analysis for upregulated and downregulated proteins. **(F)** Bubble plot was generated to visualize the top 24 signaling pathways from KEGG pathway analysis. **(G)** The expression levels of proteins associated with apoptosis, the JAK/STAT3 signaling pathway, and the cell cycle were quantified in CDN-treated versus untreated HCT116 cells. **(H)** Gene Set Enrichment Analysis (GSEA) was performed using the Gene Cards database. *n* = 3, ^*^ *p* < 0.05, ^**^ *p* < 0.01, ^***^ *p* < 0.001, vs. control group.

To further elucidate the mechanism of CDN-induced apoptosis, we performed proteomic profiling of HCT116 cells treated with CDN according to established protocols. A total of 7015 proteins were identified, each identified with at least two unique peptides at a 1% false discovery rate (FDR). Among these, 6203 proteins quantified in at least three of six biological replicates (Supplementary Table) were included in subsequent analysis. Proteomics data have been deposited at Figshare database under DOI: 10.6084/m9.figshare.30529295. Principal component analysis (PCA) showed clear separation between CDN-treated and control groups, with tight clustering within groups, indicating high reproducibility (Figure 7A). Volcano plot analysis identified 248 differentially expressed proteins (DEPs) (fold change >1.5, p < 0.05), including 108 upregulated (red) and 140 downregulated (blue) in the CDN-treated group compared to control (Figure 7B). Hierarchical clustering confirmed that the majority of DEPs exhibited reduced abundance following CDN treatment (Figure 7C). PPI network analysis using the STRING database revealed three major functional modules enriched in cell cycle regulation, JAK/STAT3 signaling, and apoptosis (Figure 7D). GO term enrichment analysis indicated that DEPs were associated with biological processes including cell cycle regulation, DNA damage response, and apoptotic processes (Figure 7E). KEGG pathway analysis highlighted apoptosis, programmed cell death, JAK/STAT3 signaling, and DNA damage response among the most significantly enriched pathways (Figure 7F). Consistent with these findings, quantitative analysis showed upregulation of apoptosis-related proteins (CASP8, CASP9, CASP3, BAX, BAD, TRADD, BIRC6) and downregulation of JAK/STAT3 signaling components (STAT3, JAK1, FOXC1, TCF12) in the CDN-treated group, notably, whereas ADAM17 and GFER were upregulated. Marked expression changes were also observed for cell cycle regulators (CDK1, CDK4, CDK6, CDKN1A) (Figure 7G). GSEA further confirmed enrichment of gene signatures related to apoptosis, p53 signaling, JAK/STAT3 signaling, and DNA damage response (Figure 7H). Together, these results demonstrated that CDN induces cytotoxicity in CRC cells primarily through JAK/STAT3 pathway-mediated apoptosis, consistent with our initial network pharmacology predictions.

### 3.8 CDN induced apoptosis in CRC cells by inhibiting the JAK/STAT3 signaling pathway

**FIGURE 8:**
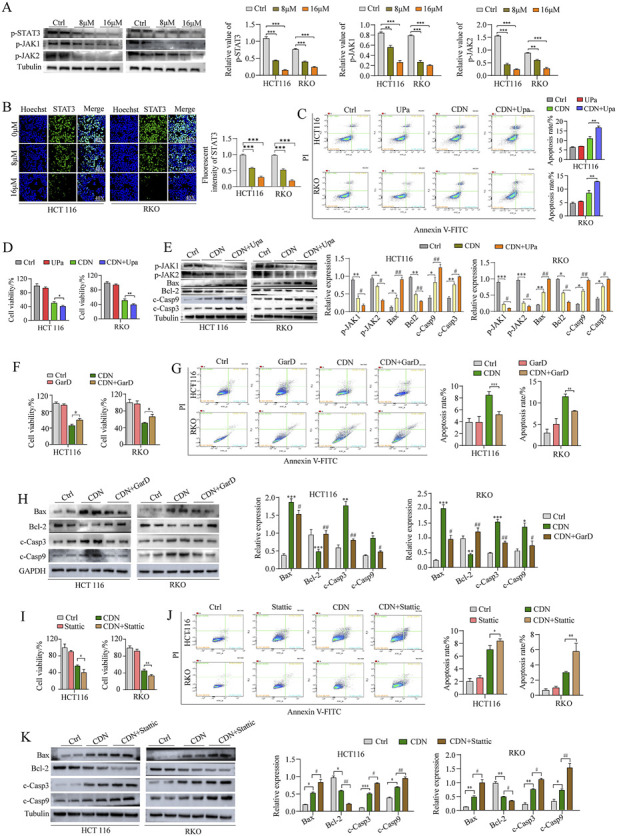
CDN induced apoptosis in CRC cells by inhibiting the JAK/STAT3 signaling pathway **(A)** Expression levels of p-JAK1, p-JAK2 and p-STAT3 were detected by Western blot analysis. **(B)** Immunofluorescence assay was used to detect the fluorescent signal of p-STAT3. **(C,D)** Cells were treated with CDN, Upa, or their combination (CDN + Upa). The apoptotic ratio and cell viability were determined, respectively. **(E)** Cells were treated with CDN alone or CDN + Upa. Western blot analysis was used to detect the expression levels of p-JAK1, p-JAK2, Bax, Bcl-2, and cleaved Casp3/9. **(F,G)** Cells were treated with CDN, GarD, or their combination (CDN + GarD). Cell viability and apoptotic ratio was determined, respectively. **(H)** Cells were treated with CDN alone or CDN + GarD. Expression levels of Bax, Bcl-2, and cleaved Casp 3/9 were quantified using Western blot. **(I,J)** Cells were treated with CDN, Stattic, or their combination (CDN + Stattic). Cell viability and apoptotic ratio was determined, respectively. **(K)** Expression levels of Bax, Bcl-2, and cleaved Casp 3/9 were quantified by Western blot. *n* = 3, ^*^ *p* < 0.05, ^**^ *p* < 0.01, ^***^ *p* < 0.001, vs. control group. ^#^ *p* < 0.05, ^##^ *p* < 0.01, vs. CDN-treated group.

Following a multi-dimensional analysis, subsequent *in vivo* experiments were performed to validate the therapeutic potential of targeting the JAK/STAT3 signaling pathway. Western blot analysis showed that CDN treatment caused a significant, dose-dependent decrease in the phosphorylation levels of JAK1 (at Tyr1022/Tyr1023), JAK2 (at Tyr1007/Tyr1008), and STAT3 (at Tyr705) (Figure 8A). These findings were corroborated by immunofluorescence assays, which yielded consistent results (Figure 8B). Notably, the combination of CDN and Upadacitinib (Upa), a JAK1/2 inhibitor, exhibited a synergistic effect: it markedly promoted apoptosis, reduced cell viability, and further decreased the expression of phosphorylated JAK1/2 (Figures 8C–E). Moreover, compared with CDN alone, the combination treatment led to increased expression of the pro-apoptotic proteins Bax and cleaved caspase-3/9, along with a significant reduction in the anti-apoptotic protein Bcl-2 (Figure 8E).

Subsequently, HCT116 and RKO cells were treated with both CDN and GarD, a STAT3-specific agonist. In comparison to CDN monotherapy, the combination resulted in a significant increase in cell viability and a marked decrease in apoptosis (Figures 8F,G), suggesting that STAT3 activation counteracts the pro-apoptotic effect of CDN. Consistent with these observations, Western blot analysis showed that GarD co-treatment reversed CDN-induced alterations in apoptosis-related proteins (Figure 8H). In contrast, combining CDN with Stattic, a STAT3-specific inhibitor, produced the opposite effect to that of GarD (Figures 8I–K). Collectively, these findings provide compelling evidence that CDN suppresses colorectal cancer by inhibiting the JAK/STAT3 signaling pathway.

### 3.9 CDN inhibited tumor growth by targeting the JAK/STAT3 signaling pathway with a favorable biosafety profile

**FIGURE 9:**
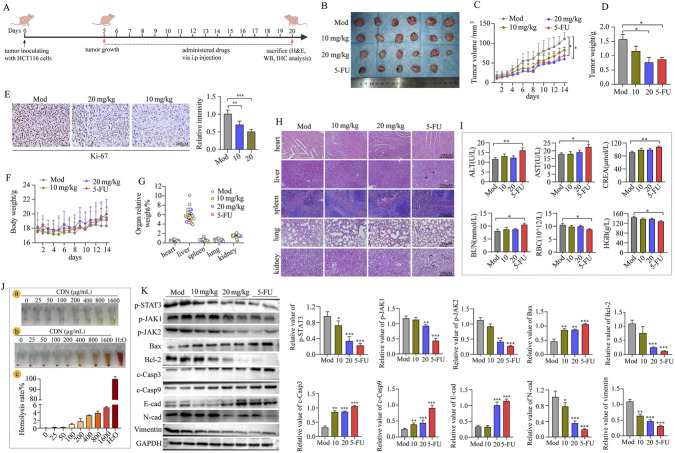
CDN suppresses tumor growth and induces apoptosis in CRC-bearing nude mice by inhibiting the activation of the JAK/STAT3/EMT signaling axis **(A)** Schematic diagram of the subcutaneous tumor transplantation protocol in nude mice. **(B)** Representative tumor images from mice in different treatment groups. **(C)** Tumor volume of each mouse was measured daily during the treatment period. **(D)** Statistical analysis of tumor weight at the experimental endpoint. **(E)** Immunohistochemical (IHC) staining of Ki-67 in tumor sections and analysis of staining intensity. Scale bar = 200 μm. **(F)** Monitoring of the body weight of mice in each treatment group throughout the study. **(G)** The relative weight of the heart, liver, spleen, lung, and kidney was calculated using the formula: Relative weight = (organ weight/mouse body weight) × 100%. **(H)** H&E staining of major organs (heart, liver, spleen, lung, and kidney tissue) for histopathological evaluation. Scale bar = 200 μm. **(I)** Detection of plasma biochemical indices and blood routine parameters. **(J)** Analysis of CDN-induced red blood cell hemolysis. (i) Observation of the background color in CDN solutions with different concentrations. (ii) Quantitative analysis of red blood cell hemolysis rate following CDN treatment. (iii) Quantitative analysis of CDN’s effect on red blood cell hemolysis. **(K)** Western blot analysis was performed to detect the relative expression levels of target proteins in tumor tissue from different treatment groups. *n* = 3, ^*^ *p* < 0.05, ^**^ *p* < 0.01, ^***^ *p* < 0.001, vs. control group.

The anti-tumor efficacy of CDN was evaluated in a subcutaneous HCT116 xenograft model (Figure 9A). CDN at 20.0 mg/kg significantly inhibited tumor growth, with an anti-tumor effect comparable to that of 5-FU (20.0 mg/kg). A trend toward decreased tumor weight and volume was also observed in the 10.0 mg/kg CDN group (Figures 9B–D). Immunohistochemical (IHC) analysis further demonstrated that CDN treatment resulted in a dose-dependent decrease in Ki-67-positive cells (Figure 9E). Biosafety assessments indicated a favorable toxicity profile for CDN. No significant differences in body weight (Figure 9F) or relative organ weights (heart, liver, spleen, lungs, and kidneys) were found between CDN-treated and model groups (Figure 9G). H&E staining confirmed the absence of pathological alterations in these organs (Figure 9H). Plasma levels of hepatic (ALT, AST) and renal (CREA, BUN) function markers in CDN-treated mice were comparable to those in the model group (Figure 9I). In contrast, 5-FU treatment induced marked toxicity, characterized by hepatic and renal vacuolation, widened renal septa (Figure 9H), significantly increased levels of ALT, AST, CREA, and BUN, and decreased red blood cell count and hemoglobin levels (Figure 9I). Furthermore, CDN showed negligible hemolytic activity, with a hemolysis rate of only 4.9% at the maximum concentration (1600 μg/mL), which is below the 5% safety threshold (Figure 9J). Consistent with our *in vitro* findings, Western blot analysis of tumor tissues revealed that CDN treatment downregulated p-JAK1/2 and p-STAT3 expression while upregulating the pro-apoptotic proteins Bax, cleaved caspase-3, and cleaved caspase-9. Additionally, CDN increased E-cadherin expression and decreased N-cadherin and vimentin levels, indicating that CDN suppresses EMT (Figure 9K). In summary, these *in vivo* results demonstrate that CDN effectively inhibits tumor growth by targeting the JAK/STAT3 pathway to promote apoptosis and suppress EMT, while maintaining a favorable biosafety profile.

## 4 Discussion

CRC continues to pose a formidable challenge in clinical oncology, particularly as the majority of patients are diagnosed at intermediate or advanced stages. This late diagnosis significantly limits therapeutic options and contributes to unsatisfactory 5-year overall survival rates. Although chemotherapeutic agents such as 5-FU and oxaliplatin can achieve transient tumor remission, their clinical benefits are often undermined by high recurrence rates and the development of primary chemoresistance, which collectively compromise long-term treatment efficacy (Li M. et al., 2024). Moreover, long-term chemotherapy is associated with severe adverse effects—including myelosuppression, hepatic and renal dysfunction, and significant weight loss—that not only diminish treatment efficacy but also considerably impair patients’ quality of life. Notably, bioactive components derived from Traditional Chinese Medicine (TCM) have shown potential in mitigating such chemotherapy-related side effects (Tian et al., 2020; Yu et al., 2023). Given these multifaceted challenges, there is an urgent need to develop novel therapeutic agents that combine high antitumor efficacy with a favorable safety profile to improve outcomes in CRC management.

**FIGURE 10:**
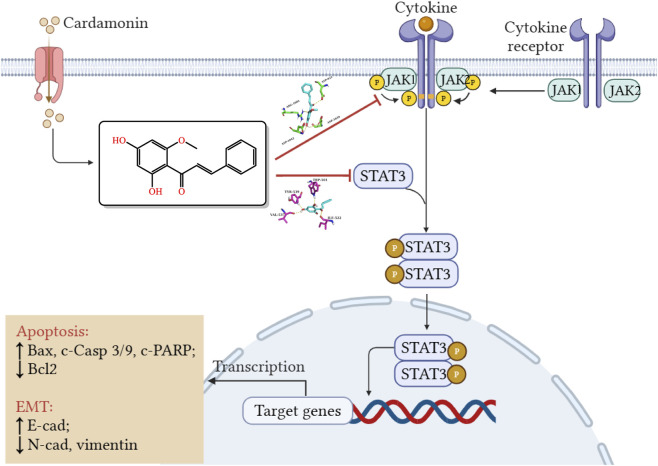
Schematic model illustrating the antitumor mechanism of CDN in CRC via inhibiting the JAK/STAT3/EMT signaling axis.

Natural compounds represent a promising source of anticancer agents due to their diverse pharmacological activities and generally favorable safety profiles (Aanniz et al., 2024; Asghar et al., 2024; Liu et al., 2024). In this study, we systematically investigated the anti-CRC mechanisms of CDN using a multi-scale approach integrating network pharmacology, proteomic profiling, molecular docking, and *in vitro/in vivo* functional assays. We found that CDN suppresses proliferation, migration, invasion, and EMT in CRC cells by inhibiting the JAK/STAT3/EMT signaling axis, ultimately promoting apoptosis and attenuating EMT (Figure 10). Compared to existing JAK inhibitors, CDN offers several advantages. First, it concurrently targets JAK1/2 and STAT3, thereby mitigating compensatory activation often seen with single-target inhibitors. Second, as a naturally derived small molecule, CDN exhibits an improved safety profile; our *in vivo* and *in vitro* studies revealed no significant hepatorenal toxicity, hematologic toxicity, organ damage, or body weight loss-side effects commonly associated with synthetic JAK inhibitors. Third, CDN exerts multi-faceted anti-CRC effects, including suppression of EMT, induction of cell cycle arrest, and inhibition of migration and invasion.

Dysregulation of the JAK/STAT3 signaling pathway is a well-established driver of CRC pathogenesis, promoting tumor progression and metastasis through enhanced proliferation, anti-apoptotic effects, and increased invasiveness (Pennel et al., 2024; Wang and Sun, 2014). This pathway promotes tumor progression and metastasis through multiple mechanisms, including enhancing tumor cell proliferation, inducing anti-apoptotic phenotypes, and strengthening invasive capabilities, positioning it as a critical regulatory axis in CRC. While previous research indicated that CDN selectively inhibits JAK2-but not JAK1-in prostate cancer models (Zhang et al., 2017). Our study demonstrates that CDN concurrently suppresses both JAK1 and JAK2 activity in CRC cells. This difference may arise from tissue-specific variations, such as distinct genetic backgrounds or differential expression of JAK/STAT3 regulators between prostate cancer and CRC. Furthermore, combining CDN with the JAK1/2 inhibitor upadacitinib synergistically enhanced apoptosis and suppressed proliferation in CRC cells, supporting the functional relevance of JAK1/2 dual inhibition.

The IL-6 and IL-11 cytokines are well-characterized upstream activators of the JAK/STAT3 signaling axis. Their engagement promotes tumor proliferation, invasion, and metastasis while contributing to immunosuppression in the tumor microenvironment (Felcher et al., 2022; Kureshi and Dougan, 2025). Analysis of the TCGA database revealed significantly higher expression of IL6 and IL11 in CRC tumor tissues compared with adjacent normal samples. Survival analyses further indicated that elevated expression of IL6, IL11, and their corresponding receptors (IL6R and IL11RA) was associated with poor prognosis in CRC patients. Similar trends were observed for JAK1 and STAT3, with high STAT3 expression significantly correlated with advanced TNM stage and reduced survival, supporting its relevance as a therapeutic target. These findings were further corroborated by single-cell sequencing data, which confirmed activation of the JAK/STAT3 signaling axis in CRC.

This study has several limitations that should be addressed in future work. First, although the inhibitory effect of CDN on the JAK/STAT3 pathway was confirmed *in vitro* and *in vivo*, its long-term pharmacokinetic profile, metabolic stability, and bioavailability remain to be fully characterized. Further preclinical ADMET studies and dose-escalation safety assessments will be essential to support the translational potential of CDN. Second, while the antitumor efficacy of CDN was demonstrated in a subcutaneous xenograft model, its interaction with the tumor immune microenvironment warrants deeper investigation, ideally using patient-derived xenograft (PDX) models that better recapitulate human tumor biology.

## Data Availability

The datasets presented in this study can be found in online repositories. The names of the repository/repositories and accession number(s) can be found in the article/Supplementary Material.

## Glossary

ALT: alanine aminotransferase
AST: aspartate aminotransferase
BUN: blood urea nitrogen
CREA: creatinine
CRC: colorectal cancer
CDN: cardamonin
FASP: filter-aided proteome preparation
FOT: fraction of total
H&E: Hematoxylin-eosin
iBAQ: intensity based absolute quantification
IC50: half maximal inhibitory concentration
IHC: immunohistochemistry
ISO: international organization for standardization
LC-MS/MS: liquid chromatography tandem mass spectrometry
MMP: mitochondrial membrane potential
ROS: reactive oxygen species
LDH: lactic dehydrogenase
DDA: data-dependent acquisition
AGC: automatic gain control
HCD: higher-energy collision dissociation
NCE: normalized collision energy
FDR: false discovery rate
GO: Gene Ontology
PPI: protein-protein interaction
PVDF: polyvinylidene fluoride
PCA: principal component analysis
DEPs: differentially expressed proteins
KEGG: Kyoto Encyclopedia of Genes and Genomes
GSEA: Gene Set Enrichment Analysis
TCGA: The Cancer Genome Atlas
OS: overall survival
scRNA-seq: single-cell RNA sequencing
Upa: Upadacitinib
GarD: Garcinone D
EMT: epithelial-mesenchymal transition
PDXs: patient-derived xenografts
